# Hydrogen-adduction to open-shell graphene fragments: spectroscopy, thermochemistry and astrochemistry

**DOI:** 10.1039/c6sc03787a

**Published:** 2016-09-26

**Authors:** Gerard D. O'Connor, Bun Chan, Julian A. Sanelli, Katie M. Cergol, Viktoras Dryza, Richard J. Payne, Evan J. Bieske, Leo Radom, Timothy W. Schmidt

**Affiliations:** a School of Chemistry , UNSW Sydney , NSW 2052 , Australia . Email: timothy.schmidt@unsw.edu.au ; Tel: +61 439 386 109; b School of Chemistry , The University of Sydney , Sydney , New South Wales 2006 , Australia; c Graduate School of Engineering , Nagasaki University , Bunkyo 1-14 , Nagasaki 852-8521 , Japan; d School of Chemistry , The University of Melbourne , Victoria 3010 , Australia

## Abstract

We apply a combination of state-of-the-art experimental and quantum-chemical methods to elucidate the electronic and chemical energetics of hydrogen adduction to a model open-shell graphene fragment. The lowest-energy adduct, 1*H*-phenalene, is determined to have a bond dissociation energy of 258.1 kJ mol^–1^, while other isomers exhibit reduced or in some cases negative bond dissociation energies, the metastable species being bound by the emergence of a conical intersection along the high-symmetry dissociation coordinate. The gas-phase excitation spectrum of 1*H*-phenalene and its radical cation are recorded using laser spectroscopy coupled to mass-spectrometry. Several electronically excited states of both species are observed, allowing the determination of the excited-state bond dissociation energy. The ionization energy of 1*H*-phenalene is determined to be 7.449(17) eV, consistent with high-level W1X-2 calculations.

## Abstract 2

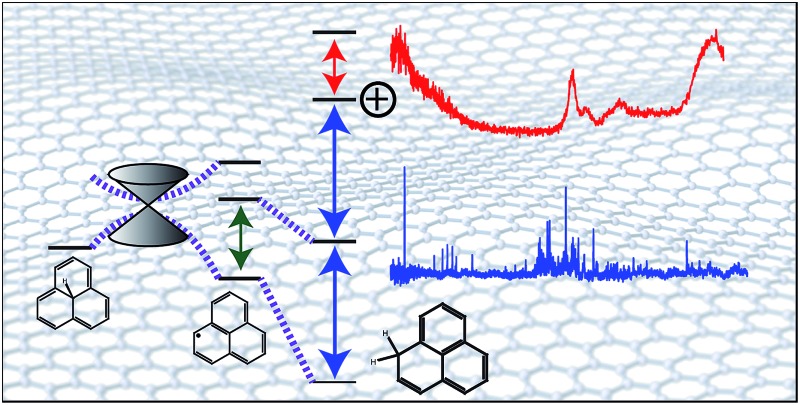
H-Adducted graphene fragments are interrogated with lasers, revealing excited state bond dissociation energies and ionization energies.

## 1 Introduction

Graphene is a material consisting of a single layer of carbon atoms bonded in a hexagon lattice.^1,2^ Its unhybridized p_*z*_ orbitals conjugate to bring about a structure with fascinating electronic properties. Interest in this material has led to an explosion of research over the past decade due to graphene's many potential applications.^1^

Addition of hydrogen to graphene alters the delocalised π-structure, removing the now sp^3^ hybridised carbon from the π-system, modifying the electronic and magnetic properties.^2–14^ The binding energy of a single hydrogen atom on graphene is reported to be just 0.7 eV.^15^ When graphene is saturated with hydrogen, one obtains the two-dimensional hydrocarbon graphane, an insulator.^3,5^ Novoselov and Geim showed that graphene hydrogenation to graphane is reversible,^5^ and, indeed, graphene has been investigated as a potential hydrogen storage material.^4,11,15^ Graphane's volumetric hydrogen capacity of 0.12 kg H_2_ per L exceeds the US Department of Energy target of 0.081 kg H_2_ per L for the year 2015.^3^

Very recently, it was shown that addition of a single hydrogen atom to graphene results in a measurable magnetic moment.^13^ Using density functional theory it has been calculated that semihydrogenated graphene (graphone) becomes a ferromagnetic semiconductor with a small indirect gap.^7^ It has also been shown theoretically that sporadic hydrogenation of graphene nanoribbons can forge new pathways towards carbon-based spintronics applications.^9^

The optical bandgap of graphene is also sensitive to the state of hydrogenation.^10,14^ Zhou *et al.* demonstrated that the electronic and magnetic properties of graphene can be finely tuned by hydrogenation.^8^ Haberer *et al.* showed that a tunable gap in quasi-free-standing monolayer graphene on gold can be induced by hydrogenation. The size of the gap reaches ∼1.0 eV for a hydrogen coverage of 8%.^10^

Chemisorption of hydrogen on graphene fragments is also believed to catalyse the formation of interstellar H_2_ molecules.^16^ As such, astronomical abundances of hydrogenated graphene nanoparticles are of fundamental astronomical interest.

Despite the intense interest in hydrogenation of graphene, its size is beyond the reach of chemically accurate calculations. However, smaller relevant systems are accessible to such accurate calculations, and these may be used to benchmark more approximate quantum chemical methods.

The phenalenyl radical consists of 13 carbon atoms and 9 hydrogen atoms arranged in the form of three conjoined six-membered rings bound at a central carbon atom. It is the smallest polycyclic subunit of graphene with an internal carbon atom and has peculiar and interesting electronic properties.^17,18^ It has been invoked as a building block for single molecule molecular conductors,^19,20^ and its high stability has led to derivatives having been observed at room temperature in solution and as crystals.^21,22^

The phenalenyl radical, and its at-first paradoxically aromatic 12-π-electron cation are also excellent candidates as astronomically relevant species, as are polycyclic aromatic hydrocarbons in general.^23–28^ Indeed, the related cation C_60_^+^ has recently been confirmed to carry interstellar absorption features (diffuse interstellar bands, DIBs),^29,30^ the first and only confirmed DIB identification.

**Fig. 1:**
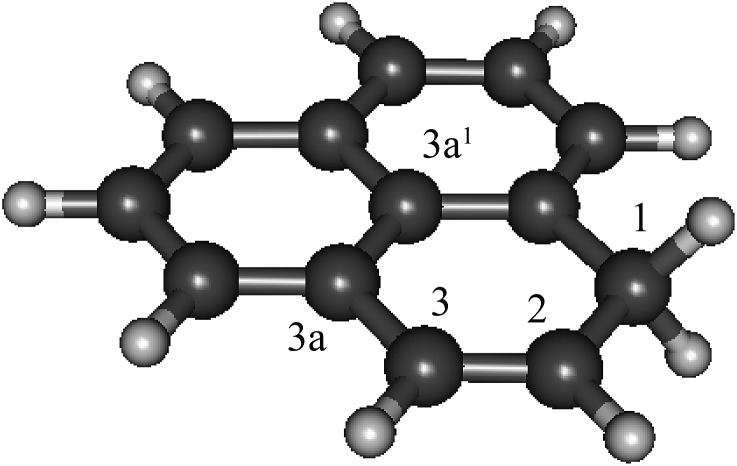
The structure of 1*H*-phenalene, the result of H-addition to phenalenyl radical (C_13_H_10_, B3-LYP/6-311G(d,p)).

As phenalenyl is the smallest scale model system of open-shell graphene nano-particles, its H-adduction product, 1*H*-phenalene (Fig. 1), may be considered a starting point in studying the electronic properties of such systems. Furthermore, substituted phenalene species have been studied as the basis of powerful organic superacids.^31^

In this work, we examine the electronic spectroscopy of the 1*H*-phenalene molecule and its radical cation. We identify two electronic states of the neutral, and three electronic states of the cation. Several vibrational assignments are made for neutral 1*H*-phenalene, which are compared with results of density functional theory calculations. We apply state-of-the-art quantum chemical methods to determine the bond dissociation energy of 1*H*-phenalene and its isomers. This is combined with spectroscopic information to determine the excited-state bond dissociation energy for 1*H*-phenalene. Our experimentally determined ionization energy is in close agreement with high-level theory. These studies form a rigorous benchmark for calculations performed with more approximate methods on much larger graphene models.

## 2 Theoretical methods

### 2.1 Geometries and frequencies

Density functional theory calculations of ground-state and excited-state geometries and vibrational frequencies were carried out at the (TD-)B3-LYP/6-311++G(d,p) level.^32–35^ Excited-state potential energy surfaces of 1*H*-phenalene were constructed by computing single-point TD-B3-LYP excited-state energies at geometries distorted by application of the appropriate excited-state vibrational coordinates. The calculated points were then fit with a cubic spline, allowing the variational calculation of (anharmonic) vibrational energy levels from the one-dimensional Schrödinger equation.^36^ The B3-LYP and TD-B3-LYP calculations were carried out using the Gaussian 09 ^37^ suite of software.

### 2.2 Vertical excitation energies

At ground-state geometries obtained by density functional theory (*vide supra*), vertical excitation energies were calculated using the X-MCQDPT2 method^38^ in the Firefly package.^39,40^ The basis sets used were of triple-zeta quality, with two sets of d-type functions for the carbon atoms and one set of p-type functions for the hydrogens. The orbitals employed were obtained at the R(O)HF level, with the active spaces as indicated in the text (*n* electrons in *o* π-orbitals, [*n*, *o*]). These calculations are taken as indicative of excitation energies, but do not account for zero-point energy differences between states or geometry relaxation in the excited state. Furthermore, the ground-state geometry is calculated at a different level of theory. This method was found previously to slightly underestimate excitation energies for a range of open-shell hydrocarbon species.^41^

### 2.3 Thermochemical calculations

Standard wavefunction and DFT calculations were carried out with the Gaussian 09 ^37^ and Molpro 2012 ^42^ programs. Geometries were optimized with the B3-LYP/6-31G(2df,p) procedure according to the G3X(MP2)-RAD protocol.^43^ Following each geometry optimization, harmonic frequency analysis was carried out to confirm the nature of the stationary point as an equilibrium structure. To obtain the zero-point vibrational energies (ZPVEs), we used B3-LYP/6-31G(2df,p) harmonic vibrational frequencies scaled by 0.9854.^44^ Refined single-point energies were obtained using a number of higher-level procedures. These included B3-LYP/6-311+(3df,3pd), G3X(MP2)-RAD,^43^ G4(MP2)-6X,^44^ CCSD(T)-F12b/VDZ,^45^ and W1X-2.^46^ The most accurate of these levels is W1X-2, which corresponds to CCSD(T) with an infinite basis set. Calculated ionization energies correspond to 0 K enthalpies, whereas bond dissociation energies are reported as vibrationless values, 0 K enthalpies, as well as 298 K enthalpies.

## 3 Experimental methods

### 3.1 Preparation of 1*H*-phenalene

1*H*-Phenalene was synthesised from commercially available perinaphthenone and reduced with diisobutylaluminum hydride in a one-step process following an established procedure.^47^

MeOH (7 mL) was cooled to 0 °C under Ar in a flask covered in foil and NaBH_4_ (140 mg, 3.6 mmol, 1.3 eq.) was added as a solid. The resulting mixture was allowed to stir for 5 min before perinaphthenone (500 mg, 2.8 mmol) was added, and the reaction allowed to warm to room temperature for 16 hours (note: exothermic, H_2_ gas released). 5% aq. HCl (0.7 mL) was added, and then the crude reaction mixture was poured onto water (50 mL) and extracted with diethyl ether (3 times, 50 mL), dried (Na_2_SO_4_) and concentrated *in vacuo* to approximately 4 mL. The crude solution was loaded directly onto a column and was purified by column chromatography (10% Et_2_O/pentane) to afford the desired compound as a white solid (125 mg, 27%). The work-up of reaction and column chromatography was carried out in the dark.

### 3.2 1*H*-Phenalene radical cation excitation spectrum

The excitation spectrum of the 1*H*-phenalene radical cation was recorded indirectly, through the predissociation spectrum of the weakly bound 1*H*-phenalene^+^···Ar complex. The spectrum of the argon-tagged cation was recorded using a tandem quadrupole–octupole–quadrupole mass spectrometer equipped with an electron-impact supersonic expansion ion source. The apparatus has been described previously.^48–52^

The 1*H*-phenalene parent sample was heated in argon behind a pulsed nozzle, seeding the supersonic expansion with 1*H*-phenalene. 1*H*-Phenalene radical cations were generated through electron impact near the nozzle orifice and clustered with Ar atoms in the free-jet expansion. The expansion was skimmed and the ions were guided into the first quadrupole mass filter by ion optics.

The first mass filter (preceding an octupole photofragmentation region) was set to *m*/*z* 206, corresponding to the 1*H*-phenalene^+^···Ar cluster, while the second mass filter was set to *m*/*z* 166, corresponding to the bare 1*H*-phenalene radical cation. Ions negotiating both the first and second quadrupole mass spectrometers were detected by a multi-channel plate. When the 1*H*-phenalene^+^···Ar clusters absorb photons and decompose, signal is observed by the increased flux of bare 1*H*-phenalene radical cations. The light source was a pulsed optical parametric oscillator (OPO) with a bandwidth of ≈8 cm^–1^. The OPO power was recorded and all reported spectra are normalized by laser power.

Note that the positions of the 1*H*-phenalene^+^···Ar absorption bands will be offset compared with the 1*H*-phenalene^+^ bands. However, these shifts have been shown to be small for similar molecules^49,53,54^ and spectra of Ar-tagged molecular cations have proved useful in determining if the observed excitation spectra are relevant to astronomical spectroscopy.

### 3.3 1*H*-Phenalene excitation spectrum

The resonance-enhanced multi-photon ionization time-of-flight (REMPI-TOF) spectrometer is identical to that used previously.^18,36,55–61^ The sample was heated in an argon atmosphere to approximately 390 K, seeding the argon with 1*H*-phenalene, behind a pulsed nozzle. The nozzle was heated to a slightly higher temperature than the sample to reduce condensation.

The pulsed nozzle was used to supersonically expand 1*H*-phenalene-seeded argon into the source chamber of the REMPI-TOF chamber. The source chamber had an operating pressure on the order of 10^–4^ Torr. The coldest part of the free jet was passed through a 2 mm skimmer into the differentially-pumped interrogation region, between the electrostatic grids of a Wiley–Mclaren time-of-flight mass spectrometer.^62^ The excitation spectrum was recorded using a combination of resonant two-photon ionization and resonant two-color two-photon ionization with Nd:YAG-pumped dye lasers.

## 4 1*H*-Phenalene radical cation excitation spectrum

**Fig. 2:**
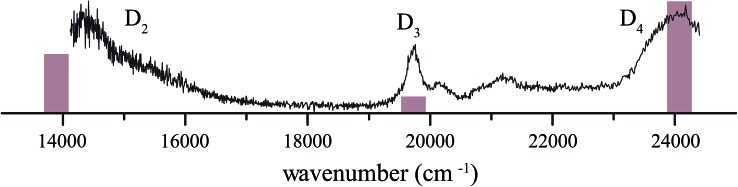
Photofragmentation spectrum of argon-tagged 1*H*-phenalene radical cation, compared with the calculated X-MCQDPT2[9,8]/tzv(2df,p) stick spectrum, shifted by +1200 cm^–1^. The increased noise at the low-energy end of the spectrum is an artefact of OPO power-correction.

The resonance-enhanced photodissociation spectrum of the 1*H*-phenalene^+^···Ar complex is shown in Fig. 2. There are a number of spectral features consistent with the existence of several electronically excited states. As an aid to the assignment of the electronic transitions, a stick spectrum of the region of interest is plotted from the X-MCQDPT2 calculated energies and (relative) intensities (Table 1). The energies for the stick-spectrum are shifted by 1200 cm^–1^ higher to allow a clearer comparison with the experimental spectrum. While the calculations were carried out for the bare radical cation, it is assumed that the energy shift due to argon-tagging is small compared with the differences in predicted energy, as in past studies.^49,52^

**Table 1:** Calculated^*a*^ X-MCQDPT2 (vertical) and experimental excitation energies (cm^–1^) of 1*H*-phenalene radical cation

State	[3, 4]	[5, 5]	[7, 7]	[9, 8]	Experiment
D_1_	10 708	8742	9341	9693	—
D_2_	19 506	11 475	12 551	12 704	14 357
D_3_	32 915	19 327	18 323	18 685	19 735
D_4_	—	30 293	21 523	21 895	23 985

^*a*^[*n*, *o*] corresponds to an active space of *n* electrons in *o* orbitals.

The 1*H*-phenalene radical cation has been previously observed in a 77 K freon matrix by Bally and co-workers.^63^ The argon-tagged 1*H*-phenalene radical cation spectrum reported in this work reproduces all the features observed in the matrix spectrum within the same spectral range. This results in amendments to the previous assignments, since any features observed in the present photofragmentation spectrum must be assigned to cationic species.

The first transition is observed as a broad strong band, which begins before the lower limit of our spectrum, centred on ∼14 360 cm^–1^ with full width at half maximum (FWHM) ∼ 1120 cm^–1^. The high-energy end of this band appears to consist of unresolved vibronic features. This is similar to the band observed, and assigned as D_2_ ← D_0_, by Bally and co-workers.^63^

The next transition observed has a Lorentzian-shaped band profile centred at about 19 735 cm^–1^ with width 290 cm^–1^. This feature was previously assigned as being carried by the neutral phenalenyl radical.^63^ This is an understandable assignment. The strongest vibronic band (*ν*_25_) of the 1^2^E′′ ← X^2^A′′_1_ (D_1_ ← D_0_) transition of the phenalenyl radical had been previously observed in this region by matrix isolation spectroscopy,^64^ and has been since recorded by us to have a gas-phase frequency of 19 560 cm^–1^.^18^ However, as neutral species such as phenalenyl radical cannot be detected by the tandem quadrupole mass-spectrometer used to record the spectrum in Fig. 2, the carrier of this band must be a cationic species with *m*/*z* ≃ 166. As such, the transition is reassigned as the electronic origin of the D_3_ ← D_0_ transition of the 1*H*-phenalene radical cation. The argument could be made that this feature is the 1^1^E′ ← X^1^A′_1_ transition of the phenalenyl (closed-shell) cation (C_13_H_9_^+^). However, this is unlikely for several reasons. The intensity of this peak correlates well with the other observed transitions, when compared with the spectrum of Bally and co-workers,^63^ and between repeated experiments. Additionally, the observed band is significantly lower in energy than our calculations suggest for the strong 1^1^E′ ← X^1^A′_1_ transition of the phenalenyl cation (23 319 cm^–1^). Indeed, the calculated 1^1^E′ ← X^1^A′_1_ transition energy of phenalenyl cation is in good agreement with the band assigned thus by Bally and co-workers, supporting this assignment. This assignment has been further supported by mass-resolved matrix spectra published by Fulara, Chakraborty and Maier, who assign a feature at 3.16 eV (25 480 cm^–1^) to the closed-shell cation, and variously calculate that this transition should lie at 3.17 eV (SAC-CI) and 3.49 eV (CASPT2).^65^ Thus, we retain the assignment of the D_3_ ← D_0_ transition of the 1*H*-phenalene radical cation for the 19 735 cm^–1^ band. The smaller features at slightly higher energy are assumed to be vibronic features of the same electronic transition.

The remaining feature is a large band at the high energy end of the scanned region. The OPO power in this region is significantly higher than for the rest of the scan. As such, to eliminate power-broadening effects, a low-power scan was performed for this region. This allowed a Lorentzian to be fitted centred on 23 985 cm^–1^ with a width of 680 cm^–1^. This peak is assigned as the D_4_ ← D_0_ transition of the 1*H*-phenalene radical cation. Despite the reduced power, this transition is still significantly saturated, and has roughly twice the intensity observed in Fig. 2 (exact quantification of the relative intensity is difficult).

## 5 1*H*-Phenalene spectrum

**Fig. 3:**
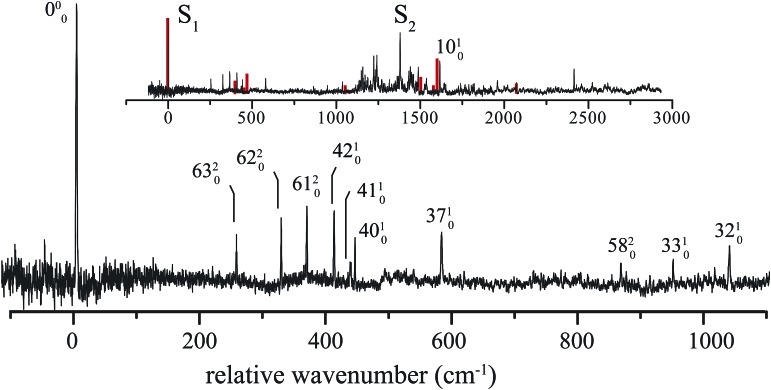
Assigned low-frequency region of the REMPI-TOF spectrum of 1*H*-phenalene (*m*/*z* 166) relative to 29 527 cm^–1^. Inset: REMPI-TOF spectrum of 1*H*-phenalene relative to 29 527 cm^–1^, compared with calculated Franck–Condon–Herzberg–Teller spectrum.

The excitation spectrum of 1*H*-phenalene is displayed in Fig. 3. The lowest-frequency band in the spectrum was observed at 29 527 cm^–1^ (353.2 kJ mol^–1^). This band is also the strongest band in the spectrum and is assigned as the S_1_ ← S_0_ electronic origin band of 1*H*-phenalene. Other features of the observed spectrum include a number of low-frequency vibrational modes, and a dense region of transitions centred about a strong band with relative frequency 1380 cm^–1^.

Due to the overwhelming number of possible assignments, an assignment of the dense region centred about 1380 cm^–1^ is not attempted in this work. However, an assignment of the relatively uncluttered low-frequency region was undertaken. This region is displayed, with assignments, in Fig. 3.

In *C*_s_ symmetry, the observed S_1_ ← S_0_ electronic transition has total symmetry A′ (2^1^A′ ← X^1^A′). Therefore, under the Franck–Condon approximation, transitions to vibrational states with total symmetry a′ will be allowed. Total symmetry a′ can be the result of any combination of in-plane a′ quanta or an even number of quanta of out-of-plane a′′ modes.

The lowest-frequency vibrational band, with a relative frequency 254 cm^–1^, is significantly lower in energy than *ν*_42_, the lowest-frequency in-plane mode. As such, this band is assigned to the two-quanta excitation of the lowest-frequency out-of-plane mode, *ν*_63_, an out-of-plane torsion of the ring containing the sp^3^ hybridised carbon. As shown in Table 2, the observed frequency of this band is bracketed by the harmonic and anharmonic calculated value. Two-quanta excitations of *ν*_62_ and *ν*_61_ are also assigned, and for these the anharmonic frequency calculations agree well, with the observed bands lying just 9 cm^–1^ and 15 cm^–1^ to higher energy than the respective calculated values. 2*ν*_60_ and 2*ν*_59_ are predicted to have relative frequencies of 486 cm^–1^ and 626 cm^–1^, respectively. No peaks are observed in these regions. However, noise around 486 cm^–1^ could possibly be hiding 2*ν*_60_. A band with relative frequency 864 cm^–1^ is close in energy to the predicted energy for two quanta of *ν*_58_ and is assigned thus.

**Table 2:** Experimental and calculated relative frequencies (cm^–1^) for vibronic bands of 1*H*-phenalene

Assignment	Exp.	Harm.	Anharm.	*Δ*
2*ν*_63_	254	224	276	+22
2*ν*_62_	325	304	316	–9
2*ν*_61_	366	384	351	–15
2*ν*_58_	864	886	884	+20
*ν*_42_	409	401	398	–11
*ν*_41_	435	420	420	–15
*ν*_40_	442	453	447	+5
*ν*_37_	580	602	602	+22
*ν*_33_	947	964		+17
*ν*_32_ ^*a*^	1036	1031		–5
*ν*_10_	1616	1603	1606	–10

^*a*^Tentative assignment.

The peak observed with relative frequency 409 cm^–1^ is assigned to the lowest-frequency in-plane a′ mode *ν*_42_, with a calculated anharmonic frequency of 398 cm^–1^. Similar assignments are made for modes *ν*_41_, *ν*_40_, *ν*_37_, *ν*_33_ and *ν*_32_. It can be seen in Table 2 that the in-plane a′ modes are relatively harmonic. As such, relatively inexpensive calculated harmonic frequencies were used for the assignment of a′ modes *ν*_33_ and *ν*_32_.

In an attempt to assign the more intense peaks at higher energies, TD-B3-LYP/6-311++G(d,p) Frank–Condon (FC) and Frank–Condon–Herzberg–Teller (FCHT) intensities were calculated.^37^ The FCHT stick-spectrum is displayed in the inset to Fig. 3.

The FCHT calculation correctly predicts the cluster of bands around 400 cm^–1^, supporting the assignment of the observed bands to single quanta of modes *ν*_42_, *ν*_41_ and *ν*_40_. The FC, FCHT and anharmonic calculations all suggest that *ν*_10_ should be strong, and this is assigned to the strong band observed at 1616 cm^–1^. However, it should be noted that TD-DFT calculations fail to reproduce the correct ordering of electronic states for the related chromophore, naphthalene. In the present chromophore, with reduced symmetry, the results of TD-DFT should be treated with caution. Nevertheless, the band at 1036 cm^–1^ is closest in energy to the calculated harmonic frequency of *ν*_32_. However, *ν*_31_ is predicted to be more intense in the FCHT simulation. The original assignment of *ν*_32_ is tentatively retained.

None of the simulations predict a strong transition at 1380 cm^–1^, or indeed any strong vibronic activity in the entire 1200–1500 cm^–1^ region. Due to the congestion in this region, combination bands are required to explain the sheer number of observed peaks. As such, there are many possible assignments in this region. The intensity of the peak at 1380 cm^–1^ is suggestive of a progression-forming mode, but there is no evidence of an overtone.

The calculated vertical S_*n*_ ← S_0_ excitation energies are given in Table 3. The calculations, which account for static correlation using an [*n*, *o*] active space of *n* electrons in *o* π-orbitals, and dynamic correlation using perturbation theory, predict a lowest excitation which is about 1000 cm^–1^ too high, an acceptable discrepancy. The calculations also clearly predict that the second excited state, S_2_ should be near-degenerate with S_1_. In the light of these calculations, we ascribe the feature at 1380 cm^–1^ to the origin of the S_2_ ← S_0_ transition, with the associated thicket of features due to vibronic coupling. The calculated transition dipole moments of these states are not orthogonal, but are disposed at an angle of 33°, indicating that the adjacent double-bond of 1*H*-phenalene results in an admixture of the L_a_ and L_b_ states of the related naphthalene chromophore.

**Table 3:** Calculated X-MCQDPT2 (vertical) and experimental excitation energies (cm^–1^) of 1*H*-phenalene

State	[4, 4]	[6, 5]	Experiment
S_1_	28 457	28 523	29 527
S_2_	30 027	30 261	30 907
S_3_	39 828	38 563	—

## 6 1*H*-Phenalene ionization energy

While recording the one-laser two-photon excitation spectrum, no bands were observed below 30 107 cm^–1^ (*ν*_37_, relative frequency 580 cm^–1^). When the 206 nm ionization laser was introduced, lower-frequency bands could be observed.

It is proposed that 29 970 cm^–1^ photons (the energy of *ν*_40_, the next highest energy band) are of insufficient energy to ionize 1*H*-phenalene from the state of the same energy. The IE of 1*H*-phenalene can thus be bracketed between twice the energies of the *ν*_37_ and *ν*_40_ bands, 2 × 29 970 < IE < 2 × 30 107 cm^–1^, 7.449(17) eV.

We previously demonstrated an approximate method for computationally bracketting the IE of resonance-stabilized hydrocarbon radicals (RSRs).^59^ For the molecules examined, IEs calculated with the B3-LYP/6-311++G(3df,3pd) and G3X(MP2)-RAD levels of theory were found to bracket the experimental figure for the range of RSRs tested. It is of interest to determine if this approximation also holds for closed-shell molecules such as 1*H*-phenalene. The IE of 1*H*-phenalene was calculated using several computational methods, as reported in Table 4. As with our previous studies on radicals, B3-LYP/6-311++G(3df,3pd) and G3X(MP2)-RAD indeed bracket the experimental result, with the highest-level W1X-2 IE of 7.49 eV close to the experimental value of 7.449(17) eV. The related W1X-1 method, when applied to the cyclohexadienyl radical, predicts an IE which is 20 meV too high, a similar discrepancy to the present case,^66–68^ noting that the experimental IE could lie anywhere within the stated range.

**Table 4:** Calculated and experimental ionization energy (IE) of 1*H*-phenalene^*a*^

Method	eV
B3-LYP/6-311++G(3df,3pd)	7.13
G3X(MP2)-RAD	7.56
G4(MP2)	7.41
G4(MP2)-6X	7.61
CCSD(T)-F12b/A'VDZ	7.44
W1X-2	7.49
Experiment	7.449(17)

^*a*^Experiment brackets result between 7.432 and 7.466 eV.

## 7 1*H*-Phenalene bond dissociation energy

The hydrogens are weakly bonded to the sp^3^ carbon of 1*H*-phenalene, due to the remarkable resonance-stability of the phenalenyl radical. The reported bond dissociation energy (BDE) is just 272(8) kJ mol^–1^.^69^ We have determined the BDE at 0 and 298 K at a range of levels of theory (Table 5). Our most accurate level of theory for all the BDEs is W1X-2, and we find also that G3X(MP2)-RAD and CCSD(T)-F12b/DVZ are generally in good agreement with W1X-2. The best calculations place the BDE close to the experimental figure, which was determined at much higher temperatures.

**Table 5:** Bond dissociation energies (BDE, kJ mol^–1^) for isomers of phenalene (C_13_H_10_)

	Vibrationless	0 K	298 K
**BDE (1*H*)**			
B3-LYP/6-311++G(3df,3pd)	271.2	239.6	244.8
G3X(MP2)-RAD	286.8	255.1	260.3
G4(MP2)	299.4	267.7	272.9
G4(MP2)-6X	310.0	278.3	283.5
CCSD(T)-F12b/VDZ	288.4	256.7	261.9
W1X-2	289.8	258.1	263.3

**BDE (2*H*)**			
B3-LYP/6-311++G(3df,3pd)	47.9	27.7	31.6
G3X(MP2)-RAD	68.5	48.3	52.1
G4(MP2)	82.2	62	65.8
G4(MP2)-6X	84.5	64.3	68.1
CCSD(T)-F12b/A'VDZ	60.3	40.1	43.9
W1X-2	58.7	38.5	42.4

**BDE (3a*H*)**			
B3-LYP/6-311++G(3df,3pd)	–16.8	–41.5	–36.7
G3X(MP2)-RAD	19.2	–5.5	–0.7
G4(MP2)	32.7	8	12.8
G4(MP2)-6X	36.4	11.7	16.5
CCSD(T)-F12b/A'VDZ	10.1	–14.6	–9.7
W1X-2	6.3	–18.4	–13.6

**BDE (3a** ^**1**^ ***H*)**			
B3-LYP/6-311++G(3df,3pd)	1.7	–38.9	–33.6
G3X(MP2)-RAD	–60.9	–101.5	–96.3
G4(MP2)	–47.4	–87.9	–82.7
G4(MP2)-6X	–40.1	–80.7	–75.4
CCSD(T)-F12b/A'VDZ	–58.8	–99.3	–94.1
W1X-2	–59.7	–100.3	–95.1

**Fig. 4:**
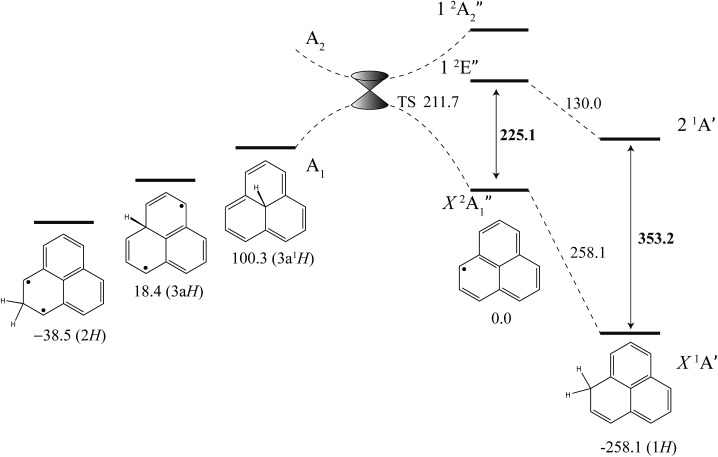
Energies of 1*H*-phenalene and its isomers (kJ mol^–1^), relative to the phenalenyl radical, calculated at the W1X-2 level (Table 5). Bold numbers are spectroscopically derived from this and previously published work.^18^

The excited-state BDE can also be determined by combining the calculated ground-state BDE with the excitation energies of the neutral phenalenyl radical^18^ (18 800 cm^–1^, 225.1 kJ mol^–1^) and 1*H*-phenalene (29 527 cm^–1^, 353.2 kJ mol^–1^), as shown in Fig. 4. Based on the 0 K W1X-2 calculations, the BDE of 1*H*-phenalene reduces from 258.1 kJ mol^–1^ to 130.0 kJ mol^–1^ in the S_1_ (2^1^A′) excited state.

Addition of hydrogen at other positions of phenalenyl radical results in phenalenes with much reduced, and in some cases negative, BDEs. The BDE of 2*H*-phenalene is calculated to be just 38.5 kJ mol^–1^, while 3a*H*- and 3a^1^ *H*-phenalene have negative BDEs. The reduced stabilities are due to a combination of strain, in the cases of 3a*H*- and 3a^1^ *H*-phenalene, and the biradical nature of the π-system in the cases of 2*H*- and 3a*H*-phenalene. The 12-electron periphery of 3a^1^ *H*-phenalene is classically anti-aromatic.

Despite the instabilities, these higher-energy isomers are all bound. Inspection of the highest-occupied molecular orbital of phenalenyl radical (a′′_1_) reveals radical density only at the 1-position and its symmetry-equivalents. As such, only addition to this site is expected to be barrierless. Dissociation of 3a^1^ *H*-phenalene, maintaining an A_1_ electronic state in *C*_3v_ symmetry, will result in a *D*_3h_ phenalenyl radical of A′′_2_ symmetry. This is an electronically excited state, which we calculate to lie near 20 205 cm^–1^ (241.7 kJ mol^–1^). Since the ground state of phenalenyl radical is of A′′_1_ symmetry, there will be a crossing of the potential energy curves for adiabatic dissociation in *C*_3v_ symmetry. Symmetry-breaking will allow these surfaces to form an avoided crossing either side of the actual crossing, at high symmetry, resulting in a conical intersection. The transition structure is located below this point, and is calculated to have an energy of 211.7 kJ mol^–1^ above the energy of phenalenyl radical (Fig. 4).

## 8 Astronomical relevance

The S_1_ ← S_0_ transition of 1*H*-phenalene is observed in the UV region, significantly too high in energy to be a carrier of a DIB – all of which are observed in the visible and NIR regions of the spectrum.^70,71^ Additionally, while having a strong origin transition, the strong vibrational bands would likely be visible in any spectra where the origin could be observed, and therefore 1*H*-phenalene also violates the non-correlation criteria for the DIBs.^72^

1*H*-Phenalene can be considered the smallest model system for edge addition of hydrogen to radical graphene fragments, altering the edge π-orbital structure. H-Addition to peripheral sp^2^ carbons of sufficiently larger open-shell graphene fragments will result in species with visible spectra. Given the relatively small FWHM of 1*H*-phenalene, it is likely that larger edge-H-adducted graphene fragments will have widths appropriate to the DIBs. Whether or not these larger molecules exhibit origin-dominated spectra remains to be seen. Spectroscopically, these H-adducted graphene fragments are unlikely to differ significantly from other large neutral closed-shell PAHs.^73,74^

Proton addition to aromatic frameworks remains of intrinsic astronomical interest. The recent identification of the C_60_^+^ radical cation as a carrier of DIBs has naturally fuelled speculation that related molecules may also be carriers. Indeed, charge-transfer bands of C_60_H^+^ and other metal-adducted and substituted fullerenes have been previously suggested as possible DIB carriers.^75^ The transitions observed in the visible predissociation spectrum of the 1*H*-phenalene^+^···Ar complex are all significantly too broad to be relevant to the DIBs. However, further experimental work on larger edge-protonated graphene molecules may be warranted.

## 9 Conclusions

The gas-phase spectra of the 1*H*-phenalene molecule and its radical cation have been presented for the first time. Multiple electronic states of both species were assigned with the aid of quantum chemical calculations. The previous assignment of 1*H*-phenalene radical cation in matrix isolation spectra was amended. The ionization energy of 1*H*-phenalene was determined to reasonable precision (17 meV), and the bond dissociation energy was calculated using high-level theory, allowing the excited-state bond dissociation energy to be determined from spectroscopic measurements. This combination of state-of-the-art spectroscopic and quantum chemical methods on a system both experimentally and theoretically tractable will enable insight into the interactions between hydrogen atoms and much larger open-shell graphene fragments.
